# A potential key mechanism in ascending aortic aneurysm development: Detection of a linear relationship between MMP-14/TIMP-2 ratio and active MMP-2

**DOI:** 10.1371/journal.pone.0212859

**Published:** 2019-02-22

**Authors:** Ramona Schmitt, Anke Tscheuschler, Philipp Laschinski, Xenia Uffelmann, Philipp Discher, Jana Fuchs, Maximilian Kreibich, Remi Peyronnet, Fabian A. Kari

**Affiliations:** 1 Department of Cardiovascular Surgery, University Heart Center Freiburg–Bad Krozingen, University of Freiburg, Freiburg, Germany; 2 Faculty of Medicine, University of Freiburg, Freiburg, Germany; 3 Institute for Experimental Cardiovascular Medicine, University Heart Center Freiburg—Bad Krozingen, University of Freiburg, Freiburg, Germany; Max Delbruck Centrum fur Molekulare Medizin Berlin Buch, GERMANY

## Abstract

**Objectives:**

Elevated matrix metalloproteinase-2 (MMP-2) tissue levels have been associated with ascending thoracic aortic aneurysm (aTAA). As MMP-2 activation is controlled by interactions among matrix metalloproteinase-14 (MMP-14), a tissue inhibitor of metalloproteinases-2 (TIMP-2) and Pro-MMP-2 in cell culture, this activation process might also play a role in aTAA.

**Methods:**

Via gelatin zymography we analyzed tissue levels of MMP-2 isoforms (Pro-MMP-2, active MMP-2, total MMP-2) and via enzyme-linked immunosorbent assay (ELISA,) MMP-14,TIMP-2 and total MMP-2 tissue levels in N = 42 patients with aTAA. As controls, MMP-14 and TIMP-2 aortic tissue levels in N = 9 patients undergoing coronary artery bypass surgery were measured via ELISA, and levels of MMP-2 isoforms in N = 11 patients via gelatin zymography.

**Results:**

Active MMP-2 was significantly higher in aTAA than in controls. Patients with aTAA exhibited significantly lower Pro-MMP-2 and TIMP-2 levels. Total MMP-2 and MMP-14 did not differ significantly between groups. Regression analysis revealed a linear relationship between TIMP-2 and the MMP-14/TIMP-2 ratio, as well as active MMP-2 in aTAA. Aneurysmatic tissue can be accurately distinguished from control aortic tissue (AUC = 1) by analyzing the active MMP-2/Pro-MMP-2 ratio with a cutoff value of 0.11, whereas MMP-14 and TIMP-2 roles are negligible in ROC analysis.

**Conclusion:**

A larger amount of MMP-2 is activated in aTAA than in control aortic tissue–a factor that seems to be a central process in aneurysm development. When active MMP-2 exceeds 10% compared to Pro-MMP-2, we conclude that it originates from aneurysmatic tissue, which we regard as a starting point for further studies of aTAA biomarkers. The tissue's MMP-14/TIMP-2 ratio may regulate the degree of Pro-MMP-2 activation as a determining factor, while the enzymatic activities of MMP-14 and TIMP-2 do not seem to play a key role in aneurysm development.

## Introduction

### Thoracic aortic aneurysms

Ascending thoracic aortic aneurysms (aTAA) remain an important challenge in terms of intervention time and screening methods in cardiovascular surgery. They are usually a silent disease, with the first symptom often an aortic rupture or aortic dissection—potentially deadly complications. The current indication for surgery is usually determined by the aneurysm's diameter (evidence level C) [1,2]. However, numerous studies report that aortic diameter alone does not seem to be a reliable indicator for surgery for aTAA, as some patients with an aneurysm exceeding intervention thresholds live for years without suffering an aortic dissection or rupture of their aneurysm, [3,4]. It is thus essential to accurately understand the pathogenesis of aTAA and evaluate markers revealing the risk of rupture or dissection other than aortic diameter alone.

### Matrix metalloproteinases

Matrix metalloproteinases (MMPs) are a family of human enzymes with 23 members capable of degrading components of the extra cellular matrix (ECM). They are involved in numerous physiological and pathological processes [5] and are synthesized as inactive pro-enzymes that require activation, and are regulated by their inhibitors, the tissue inhibitors of metalloproteinases (TIMPs), amongst others [6,7].

Since histological analyses of aTAA demonstrated significantly less elastin and collagen, the main contributors to aortic wall mechanical properties [8,9], in the aneurysm's aortic wall, the gelatinase MMP-2 has been associated with these aneurysms due to its active form's ability to degrade collagen types IV and V [10–12]. Pro-MMP-2, the inactive form of MMP-2, is expressed constitutively in the aortic wall and is activated by a complex mechanism leading to N-terminal cleavage of the Pro-domain. This activation process occurs through the interaction of Pro-MMP-2, MMP-14 (synonym MT1-MMP) and TIMP-2 on the cell surface, as demonstrated in cell cultures of human HT1080 fibrosarcoma and p2AHT2a cells (E1A-transfected human H4 (neuroglioma) cell line) [13,14].

Increased MMP-2 activation via the MMP-14-TIMP-2-mechanism and consecutively increased proteolysis could play an important role in aTAA pathogenesis [15]. Various studies have demonstrated increases in the mRNA of MMP-2 in aTAA, as well as increased active MMP-2 after inducing ascending aortic aneurysms or abdominal aortic aneurysms in animals [16–18]. One working group demonstrated significantly increased active MMP-2 in human aTAA [19].

However, no research has been conducted to date to show whether the protein levels of MMP-2, MMP-14 and TIMP-2 in human aTAA enable conclusions regarding MMP-2's activation mechanism in aTAA. It was therefore our aim to analyze levels of the MMP-2 isoforms Pro-MMP-2, active MMP-2, and total MMP-2, as well as MMP-14 and TIMP-2 in aTAAs, and to evaluate any relationships among MMP-14, TIMP-2, the MMP-14/TIMP-2 ratio, and active MMP-2. To differentiate our findings, we also analyzed a control group without aneurysm.

## Materials and methods

### Study design and patient characteristics

All recruited patients were enrolled in our clinical study entitled „Biomarkers of shear stress and wall tension in thoracic aortic aneurysms" (German clinical trial register-ID: DRKS00004866, https://www.drks.de). The aneurysm group consisted of N = 42 patients with aneurysm of the aortic root and/or the ascending aorta. Patients were included aged 18 to 85 years, and we made no selection regarding bicuspid and tricuspid aortic valves and valve function. Exclusion criteria were active malignancy, children or age above 85 years, any earlier stent graft intervention or prosthetic replacement on the aorta and aortic dissection of any kind.

The control group without aneurysm consisted of N = 17 patients undergoing coronary artery bypass surgery (CABG). Exclusion criteria were aneurysm of the aortic root and/or ascending aorta and all the aneurysm group's aforementioned exclusion criteria. The ascending-aorta diameter was below 40 mm in all control-group patients.

Patients’ characteristics and further clinical variables are summarized in Table 1. This study was approved by the Ethics Committee of the University of Freiburg and all patients gave their informed written consent.

**Table 1:** Patients characteristics.

Factors	Group	
**Aneurysm group**		
Number of patients		N = 41
Age [years] (mean(SD^1^))		61.0 (13.55)
Gender (%)	Female	10 (23.8)
	Male	32 (76.2)
Ascending aorta diameter [mm] (mean (SD))		53.57 (7.16)
	< 5.5 cm (%)	30 (71)
	> 5.5 cm (%)	12 (29)
CAD^2^		9 (21.4)
BMI^3^ (mean (SD))		27.91 (3.51)
Hypertension (%)		28 (66.6)
Hyperlipidemia (%)		19 (45.2)
Diabetes mellitus (%)		3 (7.1)
Connective tissue disorders	Marfan syndrome (%)	1 (2.4)
	ACTA 2^4^ mutation (%)	1 (2.4)
Medication	ACE.I^5^ (%)	17 (40.5)
	Sartane (%)	3 (7.1)
	Anticoagulant (%)	14 (33.3)
	Beta-blocker (%)	16 (38.0)
**Control group**		
Number of patients		N = 17
Age [years] (mean (SD))		65.6 (10.18)
Gender (%)	Female	6 (35.3)
	Male	11 (64.7)
Ascending aorta diameter [mm] (mean (SD))		34.14 (3.51)
BMI (mean (SD))		29.12 (3.63)
Hypertension (%)		15 (88.2)
Hyperlipidemia (%)		15 (88.2)
Diabetes mellitus (%)		5 (29.4)
Medication	ACE.I (%)	9 (52.9)
	Sartane (%)	4 (23.5)
	Anticoagulant (%)	17 (100)
	Beta-blocker (%)	12 (70.6)

^1^ standard deviation (SD);

^2^ coronary artery disease (CAD);

^3^ body mass index (BMI);

^4^ alpha actin 2 (ACTA 2);

^5^ angiotensin-converting enzyme inhibitor (ACE.I)

For statistical analysis, both—the aneurysmatic patients and those in the control group–were further subdivided according to the presence of comorbidities (hypertension, hyperlipidemia, diabetes mellitus, and anticoagulation therapy). The aneurysm group was subdivided further regarding terms of the recommended ascending aorta diameter intervention threshold [1] into two groups; an ascending aorta diameter < 5.5 cm and > 5.5 cm.

### Sample preparation and protein extraction

Aortic tissue from the anterior and posterior part of the aneurysm and aortic tissue from the central anastomosis in coronary bypass surgery, respectively, was deep frozen in liquid nitrogen immediately after resection and stored at -80°C. To extract proteins, aortic tissue was pulverized in liquid nitrogen and then supplied with ice-cold lysis buffer (50 mM Tris, 150 mM NaCl, 1% Triton X-100, pH 7.5) containing protease inhibitor P8340 (Sigma). Samples were incubated on ice for one hour, centrifuged for 15 minutes at 13.000 rpm and 4°C. The supernatant was filtered through spin-x-centrifuge filters (0.22 μm cellulose acetate, Costar) by centrifugation and pellets were re-suspended in lysis buffer, followed by incubation for 30 minutes, centrifugation, and filtration. The protein extracts were aliquoted and stored at -20°C. Protein extracts for the MMP-14-ELISA were made using the 1X Cell Extraction Buffer PTR (supplied by the ELISA Kit), supplied with protease inhibitor P8340. Extraction steps were the same as those mentioned above. The total protein concentration was determined via bicinchoninic acid assay (BCA) following the manufacturer’s instructions (Thermo Scientific Pierce BCA Protein Assay).

### Gelatin zymography

**Fig 1:**
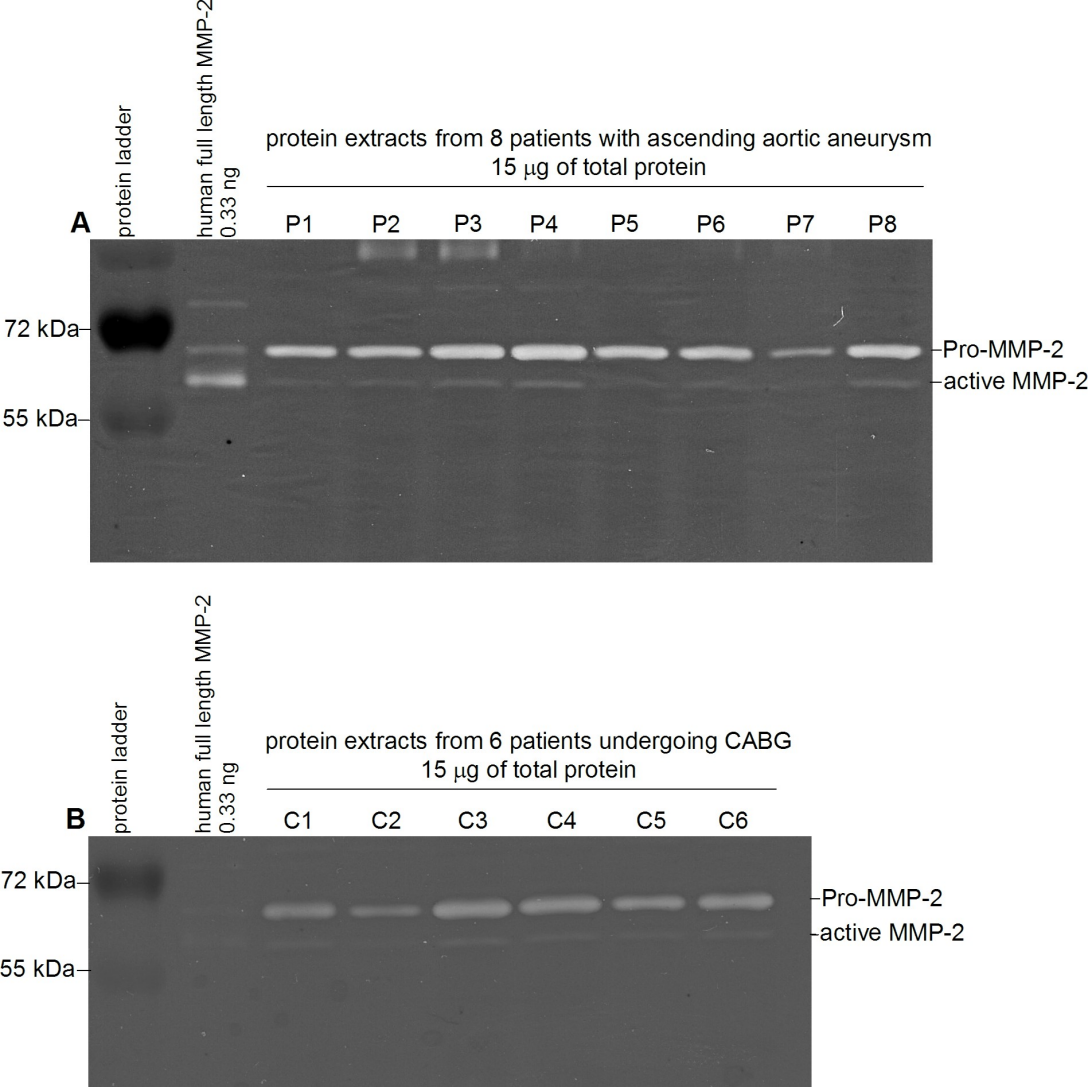
**Representative zymograms of A. 8 patients (P1-8) with ascending aortic aneurysm and B. 6 control patients (C1-6) undergoing coronary bypass surgery (CABG).** Lane 1: protein ladder. Lane 2: human full length MMP-2. Lane A. 3–10 and B. 3–8: tissue samples zymograms showing different gelatinolytic activities corresponding to Pro-MMP-2 and active MMP-2.

Gelatin zymography was carried out and validated as described previously by our group [20]. Protein extracts were diluted with zymography buffer (25mM Tris, 150mM NaCl, 10mM CaCl_2_, 0.2% Brij-35, pH 7.5) containing protease inhibitor (P8340, Sigma) and a total protein amount of 15 μg was loaded onto 8% SDS gels containing 0.2% gelatin (gelatin from porcine skin G1890, Sigma). Electrophoresis was performed at 20 mA per gel for 2.5 hours. Gels were washed twice for 30 minutes with 2.5% Triton X-100 at room temperature with agitation, followed by incubation in zymography buffer at 37.2°C for 19 hours during which gelatin digestion occurred. Afterwards, the gels were stained with 50 mL 0.2% Coomassie brilliant blue R-250 (Serva) following the manufacturer's instructions. Pro-MMP-2 and active MMP-2 in the samples were identified via a human full length MMP-2 protein (ab168864, Abcam) and were semi-quantitatively determined by analyzing pixel density with the software Image J (Version 1.47, Wayne Rasband, National Institutes of Health, USA). Each sample was normalized to 0.33 ng human full length MMP-2. All samples were measured three times independently, and those findings were averaged. MMP-2's total protein level was calculated by summarizing Pro-MMP-2 and active MMP-2 of the corresponding sample. A zymographic example is shown in Fig 1.

### Enzyme-linked immunosorbent assay (ELISA)

MMP-14, TIMP-2 and total MMP-2 tissue levels were quantified using sandwich enzyme-linked immunosorbent assay (ELISA) kits. A standard curve was run in each assay; all samples and standards were measured in duplicate and findings averaged. The TIMP-2 assay procedure was done according to the manufacturer’s instructions (DTM200 and MMP200, R&D Systems). The MMP-14 assay procedure was carried out according to the manufacturer’s instructions with the incubation time of the samples with the antibody cocktail increasing to two hours (ab197747, Abcam).

### Statistical analysis

Statistical analysis was performed using SigmaPlot Version 13.0 (Systat Software GmbH, Erkrath, Germany). Data on each aneurysm’santerior and posterior parts were averaged and tested for normal distribution by Shapiro-Wilk-Test. Furthermore, means and standard deviations were calculated. Group comparison was donevia the *t*-Test or the Mann-Whitney rank sum test. A potential linear relationship between MMP-14, TIMP-2 and active MMP-2 was tested via multiple linear regression analysis and between the MMP-14/TIMP-2 ratio and active MMP-2 via linear regression analysis. Potential differentiations of aneurysmatic from the control tissue’s protein level were obtained via receiver operating characteristic (ROC) curve and analyses of the area under the curve (AUC). Group comparison of the prevalence of comorbidities’ was conducted via χ^2^ and Fisher’s exact test. Potential correlations between ascending aorta diameter and protein levels were tested via Pearson correlation test. A <0.05 p-value was considered significant.

## Results

Our analysis of patient characteristics (Table 1) revealed that the aneurysm group's mean age was 61.0 years (standard deviation (SD) 13.55) and their mean ascending-aorta diameter measured 53.57 mm (SD 7.16). That group consisted of 10 women (23.8%) and 32 men (76.2%). The control group's mean age was 65.6 years (SD 10.18) and consisted of 6 women (35.3%) and 11 men (64.7%); their mean ascending-aorta diameter measured 34.14 mm (SD 3.51). The mean age between the two groups did not differ significantly (p = 0.212).

Regarding the patients’ comorbidities: analyses of the two groups revealed no significant difference in the prevalence of hypertension (p = 0.115). The control-group patients had hyperlipidemia (p<0.001) and diabetes mellitus (p = 0.024) significantly more often, and were significantly more often anticoagulated (p<0.001) than patients in the aneurysm group.

### Zymographic and ELISA results

Our zymographic results (S1 Table) revealed these mean protein levels: Pro-MMP-2 168.1 arbitrary units (AU) (SD 42.40), active MMP-2 30.73 AU (SD 10.38) and total MMP-2 200.13 AU (SD 46.41) in the aneurysm group. In comparison, the control group's mean protein levels were Pro-MMP-2 201.54 AU (SD 43.52), active MMP-2 13.86 AU (SD 2.94) and total MMP-2 215.40 AU (45.41).

**Fig 2:**
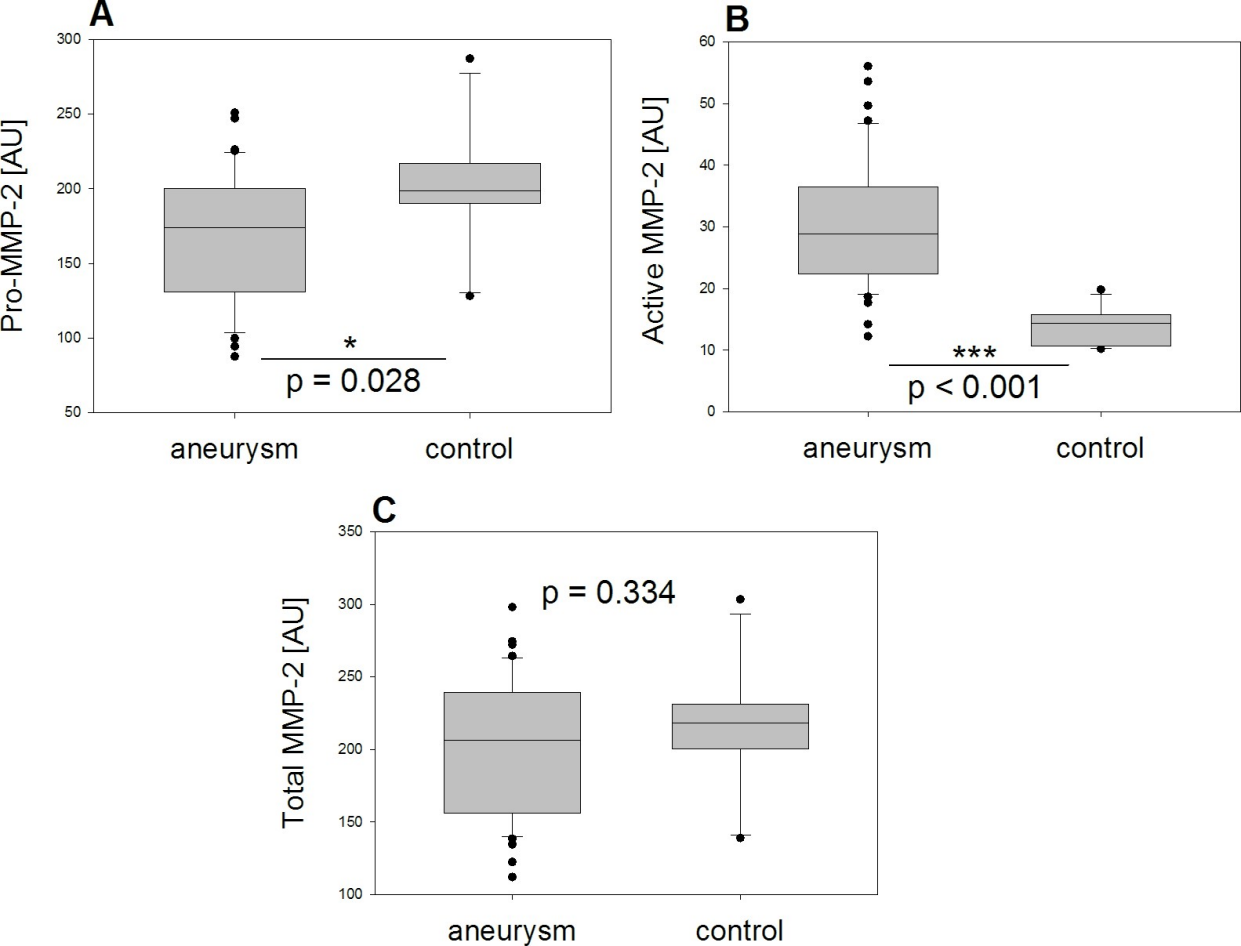
Results of zymography. Comparison between aneurysm and control group (A) Pro-MMP-2, (B) active MMP-2, (C) total MMP-2 reveals significantly larger amounts of active MMP-2 and significant smaller amounts of Pro-MMP-2 in the aneurysm group, whereas total MMP-2 did not differ significantly between the two groups *(MMP-2 isoforms given in AU)*.

Our analyses of semiquantitative measurements of the different MMP-2 isoforms by zymography (Fig 2) exhibited significantly more active MMP-2 in tissue from aTAA than in controls (p<0.001), whereas Pro-MMP-2 was significantly lower in tissue from aTAA (p = 0.028). In contrast, total MMP-2, measured by summarizing Pro-MMP-2 and active MMP-2, did not differ significantly between the two groups (p = 0.334). This result was confirmed by analysis of total MMP-2 ELISA results (aneurysm group mean 36.06 ng/ml (SD 17.43); control group mean 51.90 ng/ml (SD 28.50); p = 0.145).

Our ELISA results (S1 Table) in the aneurysm group revealed that mean MMP-14 measured 3.55 ng/mL (SD 1,22) and mean TIMP-2 22.77 ng/mL (SD 7.99), whereas in the control group mean MMP-14 measured 3.71 ng/mL (SD 0.84) and mean TIMP-2 30.94 ng/mL (12.40).

**Fig 3:**
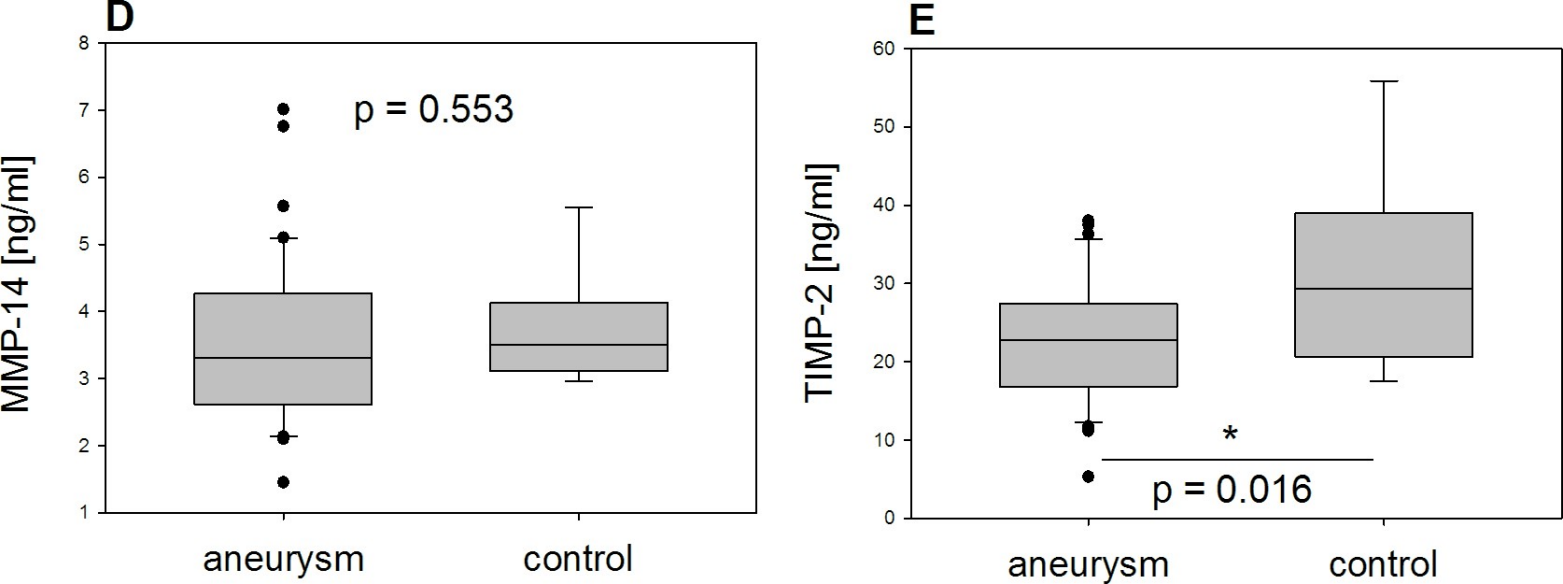
Results of ELISA. Comparison between aneurysm and control group (D) MMP-14 and (E) TIMP-2 shows significant lower amounts of TIMP-2 in the aneurysm group whereas MMP-14 did not differ significantly between the two groups *(MMP-14 and TIMP-2 given in ng/mL)*.

Our analyses of measurements by ELISA (Fig 3) revealed no significant difference in MMP-14 between the two groups (p = 0.553) whereas TIMP-2 was significantly lower in tissue from aTAA than in tissue from controls (p = 0.016).

### Regression analyses results

Based on our aneurysm group's protein levels, we conducted regression analyses to evaluate any relationships among the various proteins (S1 Fig). The p-values of the regression coefficients revealed no linear relationship between MMP-14 and active MMP-2 (r = 0.006; p = 0.997), but a linear relationship did appear between TIMP-2 and active MMP-2 (r = 0.661; p = 0.002).

Resembling the relationship between TIMP-2 and active MMP-2, the regression coefficient's p-value displayed a linear relationship between the MMP-14/TIMP-2 ratio and active MMP-2 (r = -0.434; p = 0.026).

### Analysis of ROC curve

**Fig 4:**
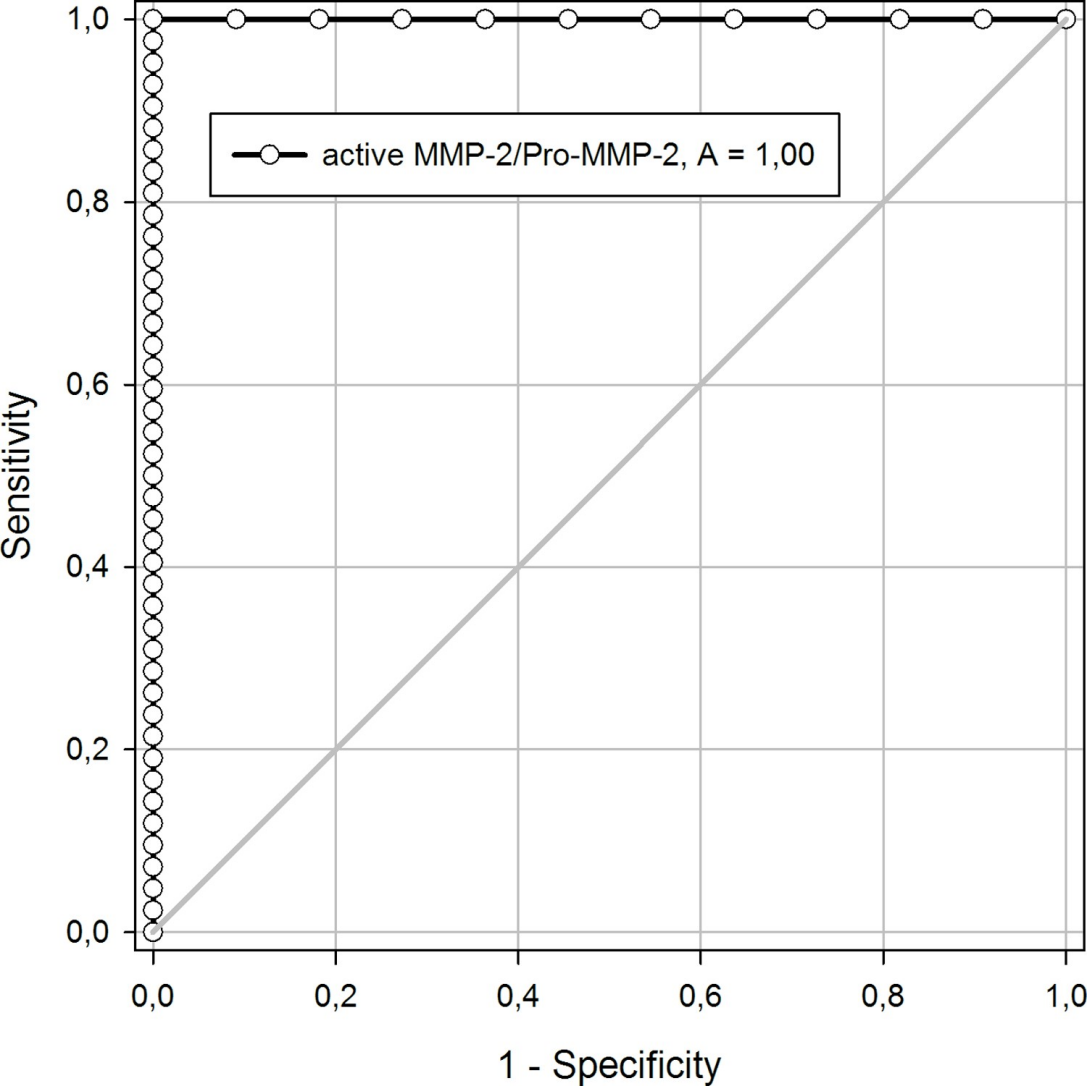
ROC curve of active MMP-2/Pro-MMP-2 ratio. The AUC (A) analyzing active MMP-2/Pro-MMP-2 ratio with a cutoff value of 0.11 illustrates perfect discrimination (AUC = 1) of aneurysmatic from control tissue.

To analyze whether aneurysmatic tissue can be discriminated from control tissue, we performed ROC curve and AUC analyses. Analyses of the ROC curves showed that the AUC of Pro-MMP-2 was 0.900 (95% Confidence Interval (CI) 0.80–1.0) and active MMP-2 was 0.996 (95% CI 0.92–1.00). We enabled perfect discrimination (AUC = 1) by analyzing active MMP-2/Pro-MMP-2 ratio applying a cutoff value of 0.11 (Fig 4).

AUC of total MMP-2 (AUC 0.576; 95% CI 0.40–0.76), MMP-14 (AUC 0.565; 95% CI 0.39–0.74), TIMP-2 (AUC 0.698; 95% CI 0.51–0.89) as well as MMP-14/TIMP-2 ratio (AUC 0.632; 95% CI 0.44.0.83) yielded no significant discrimination between aneurysmatic and control tissue.

### Association between protein levels and comorbidities

Active MMP-2 was significantly higher in the group of patients with ascending aortic aneurysm and hyperlipidemia compared to those without hyperlipidemia (p = 0.009). We detected no other association among protein levels and the comorbidities hypertension, hyperlipidemia, and diabetes mellitus, or the presence of anticoagulation therapy (Table 2).

**Table 2:** Analysis of comorbidities' influence on protein levels in the aneurysm group.

Comorbidity/Medication	Protein	p-value
Hypertension	Pro-MMP-2	0.773
	Active MMP-2	0.256
	Total MMP-2	0.290
	MMP-14	0.301
	TIMP-2	0.879
Hyperlipidemia	Pro-MMP-2	0.120
	**Active MMP-2**	**0.009**
	Total MMP-2	0.097
	MMP-14	0.990
	TIMP-2	0.247
Diabetes mellitus	Pro-MMP-2	0.412
	Active MMP-2	0.861
	Total MMP-2	0.884
	MMP-14	0.617
	TIMP-2	0.908
Anticoagulation	Pro-MMP-2	1.000
	Active MMP-2	0.267
	Total MMP-2	0.770
	MMP-14	0.699
	TIMP-2	0.457

As only hyperlipidemia revealed an association with active MMP-2, we conducted regression analyses only for the hyperlipidemic subgroup. That subgroup’s analyses as well as the subgroup’s without hyperlipidemia showed no significant linear relationship between MMP-14, TIMP-2, MMP-14/TIMP-2 and active MMP-2 (with hyperlipidemia p = 0.357 linear regression and p = 0.092 multiple regression; without hyperlipidemia p = 0.221 linear regression and p = 0.061 multiple regression).

Our control group analyses displayed no significant association between Pro-MMP-2 (p = 0.763), active MMP-2 (p = 0.291), total MMP-2 (p = 0.722), or MMP-14 (p = 0.730) and diabetes mellitus. Furthermore, MMP-14 was not associated with hypertension (p = 0.475).

### Association between protein levels and ascending aorta diameter

Correlation analyses of protein levels with ascending aorta diameter in the aneurysm group showed no significant correlation between any measured protein level and ascending aorta diameter (p>0.050 each).

Our analyses of the subdivided groups (ascending aorta diameter < 5.5 and > 5.5 cm) revealed significantly higher protein levels of Pro-MMP-2 (p = 0.032) and total MMP-2 (p = 0.030) in the aneurysms < 5.5 cm. There were no significant differences in the other proteins’ levels (active MMP-2 p = 294; MMP-14 p = 504; TIMP-2 p = 0.517).

Regression analyses showed that only the p-values of the regression coefficients in aneurysms < 5.5 cm depicted a linear relationship between active MMP-2 and TIMP-2 (p = 0.002), as well as between active MMP-2 and MMP-14/TIMP-2 ratio (p = 0.024). These relationships did not exist in aneurysms > 5.5 cm (multiple regression p = 0.272; linear regression p = 0.954).

Neither the MMP-2 isoforms nor MMP-14 or TIMP-2 exhibited a correlation with ascending aorta diameter (p>0.050 each). In the control group, regression analyses were not possible due to the small samples.

## Discussion

Our study aimed to analyze MMP-2 isoforms as well as the MMP-2 activating enzymes TIMP-2 and MMP-14 in ascending aortic aneurysm and control tissue from patients undergoing CABG.

### Study group

Regarding our patients’ comorbidities it is not surprising that patients undergoing CABG suffered more often from hyperlipidemia and diabetes mellitus, as these comorbidities are strongly associated with coronary artery disease [21]. Furthermore, anticoagulation (for example, low-dose aspirin) in patients undergoing CABG is a standard therapy [21] and thus no patient in our control group was not on anticoagulation therapy.

### Protein levels and their interactions

Our protein level results further confirm a significant increase in the amount of active MMP-2 in aneurysm of the ascending aorta, as other groups have reported [19]. Since total MMP-2 did not differ between aneurysm and controls, and Pro-MMP-2 was higher in controls, this result enables us to conclude that a larger amount of MMP-2 is activated in the aneurysm, whereas the total level of protein does not differ between aneurysm and control aortic tissue. Measurements of total MMP-2 by ELISA confirmed our zymographic results, as we observed no significant difference in total MMP-2 between the two groups in this analysis either.

Furthermore, the aneurysm group's significant increase in active MMP-2 supports the hypothesis of increased proteolysis in the aneurysm [15], which could be a determining factor in the development and progression of aTAA.

Increased active MMP-2 in the human ascending aorta of patients undergoing coronary artery bypass surgery correlated positively with older age in one study [22]. As our control group was age-matched with the aneurysm group, the increased active MMP-2 we observed is caused by a genuine increase most likely due to aneurysm formation.

Our findings enable various hypotheses regarding MMP-2 activation in aTAA. Since we noted a linear relationship between TIMP-2's protein levels and active MMP-2, as well as between the MMP-14/TIMP-2 ratio and active MMP-2, one can conclude that the activation mechanism revealed via cell culture by MMP-14 and TIMP-2 seems to play a determining role in MMP-2's activation process in human aTAA also. Although this conclusion is based on protein levels, it can be considered an indication (or at least hint) that the activation process described in a cell culture of HT1080 fibrosarcoma and p2AHT2a cells [14] also takes place in vivo in the ascending aorta. As a limiting factor, we cannot exclude other MMP-2 activation mechanisms, however, experiments in cell cultures so far have shown that these mechanisms are slower and less efficient than the MMP-14-TIMP-2 mechanism [23].

With regard to the ROC curve's analysis we conclude that wen MMP-2 activity exceeds 10% compared to Pro-MMP-2, the active MMP-2 comes from aneurysmal tissue. This perfect discrimination of aneurysmatic from control tissue with an AUC = 1 underlines the central role of MMP-2 activation in aneurysmal growth and development. Our analysis also confirms the high sensitivity and specificity of our MMP-2 isoforms’ zymographic findings.

On the other hand, MMP-14, TIMP-2 and their ratio in ROC curve are negligible compared to that of MMP-2, enabling us to hypothesize that the enzymes that activate MMP-2 are not the determining enzymatic factor in aneurysm development, rather that they support the central role of MMP-2 activation.

The perfect discrimination possible when MMP-2 activity is above 10% can also be seen as a starting point for future studies with regard to screening methods for aTAA.

TIMP-2 could be a determining factor in MMP-2 activation, whereas MMP-14 is only required in a basal amount, since TIMP-2 exhibited a linear relationship with active MMP-2, and because MMP-14 did not differ between our aneurysm and control groups. TIMP-2 seems to play a key role in MMP-2 activation, as an increase in MMP-14 does not result in increased MMP-2 activation without incurring certain TIMP-2 levels [24]. In cell culture, TIMP-2 acts as an MMP-2 activator at low protein levels whereas activation is inhibited at high protein levels. In other studies, TIMP-2 was not quantified but titrated until activation of MMP-2 was inhibited [24,25]. Thus, we cannot conclude whether our TIMP-2 levels can be ranked as low or high protein levels. However, TIMP-2 in the aneurysm could be downregulated to increase MMP-2 activation, whereas TIMP-2's inhibitory function is predominant in controls. This finding is in line with our finding that TIMP-2 was significantly higher in controls. Why might patients lose TIMP-2's inhibitory, protective function? Why is it downregulated? This might be part of a reactive local tissue mechanism counteracting enhanced local mechanical stress and strain, a mechanism that gradually becomes intensified and evolves from being protective (making the aortic wall stiffer as a reaction to enhanced stress) to being harmful (leading to a loss of elasticity and thus making dilatation and dissections more likely).

On the other hand, if our finding of lower TIMP-2 levels in aneurysm than in control tissue potentially indicates levels too low to activate MMP-2, those can be seen as a hypothesis-generating finding also. Enhanced active MMP-2 despite too low TIMP-2 could be generated by other MMP-2 activating mechanisms, for example intracellular mechanisms. Furthermore, we cannot rule out that the effects of extraction mechanism differ between aneurysmatic and control matrix. The aneurysmatic matrix could have trapped TIMP-2 during the extraction process and thus made it ELISA-inaccessible. In addition, interference in TIMP-2 assessment is caused by excessive MMP-2 tissue according to the manufacturer’s manual (reacts 0.002% at concentrations > 2.5 ng/ml). We imagine that high MMP-2 concentrations increase the binding rate to TIMP-2 and thus interfere with the antibody detection in the ELISA kit.

In further studies it will be essential to evaluate these mechanisms and potential interactions between TIMP-2 and MMP-2, as well as to analyze any significant intracellular MMP-2 activation. This could, for example, be achieved by immunohistochemistry to differentiate MMP-2 isoforms, MMP-14 and TIMP-2 intra- and extracellularly.

On the other hand, MMP-14 activity could be a determining factor in MMP-2 activation and differ between the two groups—a potential explanation for our finding that MMP-14's total protein level does not differ between the aneurysm and control groups, although MMP-14 is assigned an important role in the activation process of MMP-2 in the literature, and a cell-culture study reported no MMP-2 activation at all without the presence of MMP-14 [26]. If there are differences between total protein levels and the level of active MMP-14, it is also possible that active MMP-14 will exhibit a linear relationship with TIMP-2 and active MMP-2. MMP-14's different regulation mechanisms, for example by phosphorylation and endocytosis, are known [27,28] and these may explain differences between the level of active MMP-14 and the total protein level.

Protein levels may also differ between the early stage of aneurysm development and the time of surgery. Numerous studies have demonstrated elevated amounts of MMP-14 mRNA after inducing aneurysms in animal models [18,29]. Another group then reported subsidence in the amounts of mRNA after an aneurysm's development down to the level of a control group without aneurysm [18]. Thus, MMP-14 could be a determining factor in rising protein levels during an aTAA's initial development, and a supporting factor in basal protein levels later. This is another possible explanation for our finding that MMP-14 does not differ between aneurysms and controls at the time of surgery. However, this result stands in contrast to a study showing elevated MMP-14 in aTAA [27]. Indeed, the many differences among various study results reflect the complexity of aneurysmatic disease and of how much remains unknown about aTAA's exact pathogenesis. Therefore, future working groups will need to investigate how, precisely, MMP-14 is regulated, as well as its activity in aTAA and role in MMP-2 activation at different stages of aneurysm development.

### Comorbidities and ascending aorta diameter

Our group previously showed that hyperlipidemia may bias MMP serum levels in patients with aTAA [30]. In the present study, tissue protein levels only demonstrated an association among active MMP-2 and the prevalence of hyperlipidemia and higher levels of active MMP-2 in the group of patients with hyperlipidemia. This association should be further investigated as we have only observed an association, and thus cannot draw conclusions about the underlying pathophysiological mechanism.

A previous study showed that chronically elevated plasma glucose increases plasma MMP-2 [31]. We were unable to demonstrate any association between MMP levels and the presence of diabetes mellitus in aortic tissue. As a limiting factor, it remains unclear whether our diabetic patients constitute a group of patients with chronically elevated plasma glucose, as we did not measure glucose levels in this study. Furthermore, only three patients in the aneurysm group and five in the control group had diabetes. Therefore, we cannot conclude that there is no association between diabetes and MMP levels in a larger patient group.

Ascending aorta diameter did not correlate with any measured protein level. Nevertheless, there were significant differences between aneurysms < 5.5 cm and aneurysms > 5.5 cm in Pro-MMP-2 and total MMP-2 levels, and our regression analysis revealed linear relationships among the overall aneurysm group with aneurysms < 5.5.cm. Ascending aorta diameter hus seems to influence protein levels. This result is supported by a previous investigation that found a weak positive relation between MMP-2 and elastic modulus of the ascending aorta wall [32]. Further studies will need to specify this influence. Is it the diameter alone that changes protein levels, or do anomalies in the aortic wall matrix and in hemodynamics (due to the increasing diameter) influence the protein levels?

### Limitations

As we had access to so little aortic tissue acquired during coronary artery bypass surgery, we could not quantify every protein level in each control sample. We were thus unable to conduct regression analyses of the influence of MMP-14 and TIMP-2 on the amount of active MMP-2 in our control group. Analyses of associations with hyperlipidemia, between TIMP-2 and diabetes, as well as the association between MMP-2 isoforms and TIMP-2 with hypertension were also impossible in our control group–also due to the tiny tissue samples.

Our control group does not represent a healthy group. Coronary artery disease (CAD) has also been associated with MMP-2 [33,34]. As there were patients with CAD in the aneurysm group too, increased levels of MMPs could be determined by aneurysm and/or CAD. However, since the protein levels we measured depicted significant differences between aneurysm and CAD patients, it seems reasonable to conclude that changes in protein levels in aneurysms are not predominantly caused by the simultaneously presence of CAD.

We cannot draw conclusions about the influence of Marfan syndrome and ACTA 2 mutation on the protein levels we measured as only two patients presented connective-tissue disorders and thus meaningful statistical analysis was infeasible regarding those protein levels. Nevertheless, these disorders have been associated with abnormal MMP levels in comparison to aneurysms not caused by connective diseases [35], and our analyses should be repeated in a larger patient group.

The protein levels measured in this study only depict one endpoint of aneurysm development, the surgery timepoint. It is thus not possible to draw conclusions about earlier stages of aneurysm development and the role of MMPs at other timepoints. In addition, we cannot exclude changes in protein levels due to extraction methods–a limiting factor in all studies analyzing protein levels in tissue samples.

### Perspective

Despite such varying hypotheses, we maintain that the linear relationships between TIMP-2 and active MMP-2 and between the MMP-14/TIMP-2 ratio and active MMP-2 are interesting new findings indeed, as our ROC curve analysis underlined the potential central role of MMP-2 in ascending aneurysm development.

Specific TIMP-2/MMP-14 ratios have been investigated in an baculovirus/insect-cell expression system, and a threshold for TIMP-2's enhancing effect in MMP-2 activation (as well as a molar fraction for its maximum enhancing effect) have been detected [36]. However, no such studies have investigated such human cells in aTAA tissue.The MMP-14/TIMP-2 ratio may be a determining factor in regulating the levels of active MMP-2 in aTAA. As only TIMP-2 revealed a linear relationship with active MMP-2, one can conclude that TIMP-2 is the determining factor in this ratio. Further studies should investigate whether there is a specific ratio at which MMP-2 activation exceeds the threshold of normal activation in healthy aortic tissue, that is, when aneurysm development is initiated. Moreover, further investigation is required to evaluate whether a specific ratio (or a specific ratio's disequilibrium) correlates with the aneurysm's expansion or the risk of aortic dissection.

These questions, as well as analyses of comorbidities and ascending aorta diameter, contribute to the central issue in aTAA development, namely which influencing factors initiate their development and exacerbate them. Induced activation and increased active MMP-2 are known to occur through rising wall shear stress and excessive angiotensin 2 levels in the aneurysm amongst others [37,38]. It is unknown whether these influencing factors specifically regulate MMP-14 and TIMP-2 and therefore the MMP-14/TIMP-2 ratio in aTAA. Cell cultures have revealed a correlation between MMP-14 and wall shear stress [38].

Further investigation is required to evaluate these regulatory mechanisms in aTAA. If there is a relationship between factors influencing aTAA development and the MMP-14/TIMP-2 ratio, that could be considered a starting point for further research into identifying aTAA biomarkers and into optimizing the surgery timepoint, as wall shear stress can be monitored noninvasively in patients. As our ROC curve revealed perfect discrimination of aneurysmatic from control aortic tissue when active MMP-2 is above 10% compared to Pro-MMP-2, this can also be seen as a starting point for identifying biomarkers.

## Conclusion

Our results indicate that significantly more MMP-2 is activated in aTAA than in control aortic tissue. The central role of MMP-2 in aneurysm development is underlined by ROC analysis and the discrimination of aneurysmatic from control aortic tissue when active MMP-2 exceeds 10% compared to Pro-MMP-2 can be seen as a starting point for identifying aTAA biomarkers. The local tissue's MMP-14/TIMP-2 ratio may regulate the degree of Pro-MMP-2 activation in human aTAA as a determining factor, but MMP-14 and TIMP-2 do not seem to be enzymatically essential to aneurysm development.

## Supporting information

S1 Fig Results of regression analyses. Scatterplot (A) relationship between TIMP-2 and active MMP-2, (B) relationship between MMP-14 and active MMP-2 and (C) relationship between MMP-14/TIMP-2 ratio and active MMP-2. Scatterplots show a linear relationship between TIMP-2 and active MMP-2 as well as a linear relationship between MMP-14/TIMP-2 ratio and active MMP-2 *(MMP-2 isoforms given in AU; TIMP-2 and MMP-14 given in ng/mL)*.
(TIF)

S1 Table MMP-2, MMP-14 and TIMP-2 values used for analyses. MMP-2 values were calculated as described in Material and methods.
(XLSX)
