# Supplementary figures and images for: A potential key mechanism in ascending aortic aneurysm development: Detection of a linear relationship between MMP-14/TIMP-2 ratio and active MMP-2
Source: PLoS One. 2019 Feb 22;14(2):e0212859. doi: 10.1371/journal.pone.0212859 (PMC6386481; doi:10.1371/journal.pone.0212859)

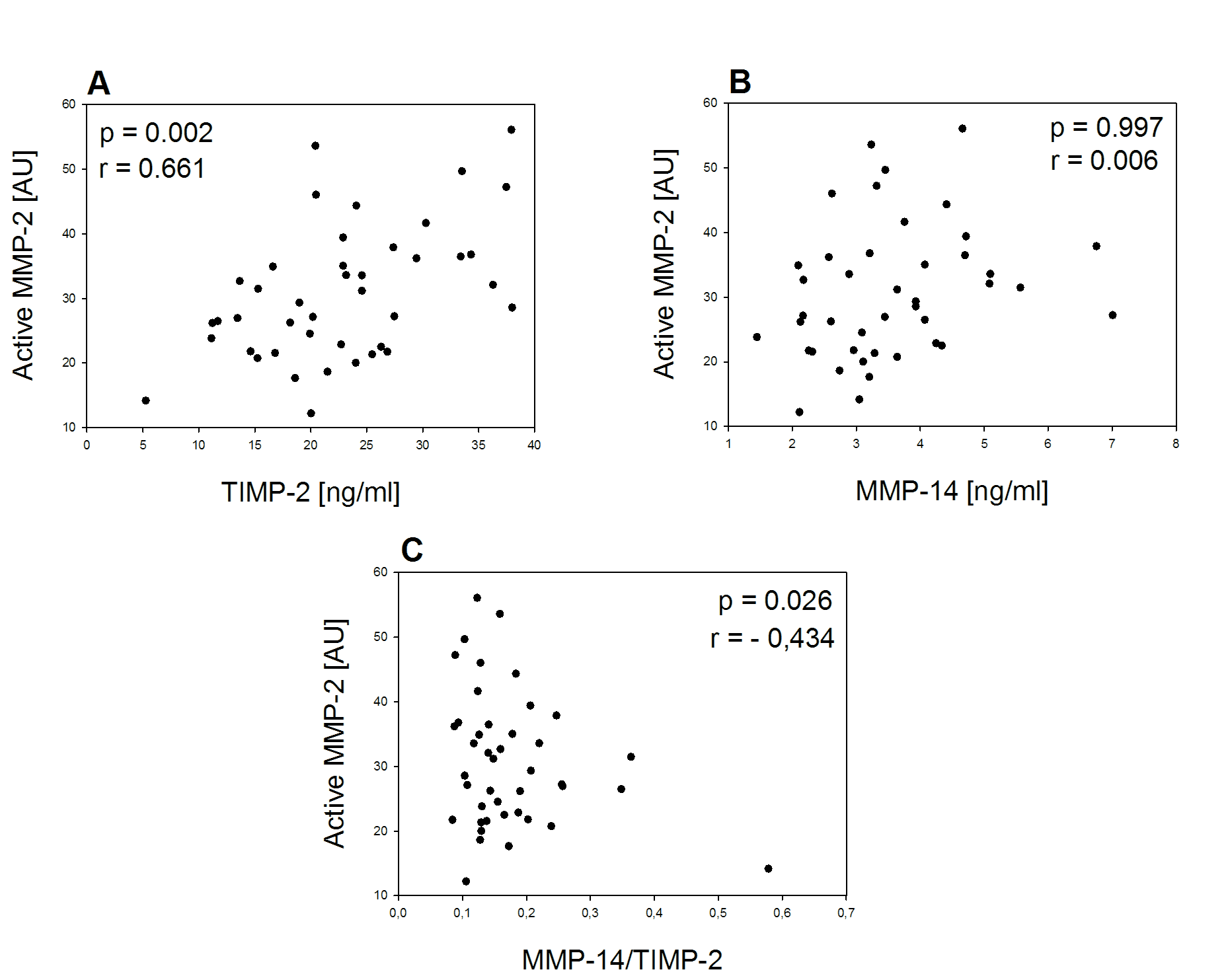

Supplement: S1 Fig — Scatterplot (A) relationship between TIMP-2 and active MMP-2, (B) relationship between MMP-14 and active MMP-2 and (C) relationship between MMP-14/TIMP-2 ratio and active MMP-2. Scatterplots show a linear relationship between TIMP-2 and active MMP-2 as well as a linear relationship between MMP-14/TIMP-2 ratio and active MMP-2 (MMP-2 isoforms given in AU; TIMP-2 and MMP-14 given in ng/mL). (TIF) [file pone.0212859.s001.tif]
